# Wnt, Hedgehog and Junctional Armadillo/β-Catenin Establish Planar Polarity in the *Drosophila* Embryo

**DOI:** 10.1371/journal.pone.0000009

**Published:** 2006-12-20

**Authors:** Pamela F. Colosimo, Nicholas S. Tolwinski

**Affiliations:** Program in Developmental Biology, Sloan-Kettering Institute, Memorial Sloan-Kettering Cancer Center New York, New York, United States of America; Cambridge University, United Kingdom

## Abstract

To generate specialized structures, cells must obtain positional and directional information. In multi-cellular organisms, cells use the non-canonical Wnt or planar cell polarity (PCP) signaling pathway to establish directionality within a cell. In vertebrates, several Wnt molecules have been proposed as permissible polarity signals, but none has been shown to provide a directional cue. While PCP signaling components are conserved from human to fly, no PCP ligands have been reported in *Drosophila*. Here we report that in the epidermis of the *Drosophila* embryo two signaling molecules, Hedgehog (Hh) and Wingless (Wg or Wnt1), provide directional cues that induce the proper orientation of Actin-rich structures in the larval cuticle. We further find that proper polarity in the late embryo also involves the asymmetric distribution and phosphorylation of Armadillo (Arm or β-catenin) at the membrane and that interference with this Arm phosphorylation leads to polarity defects. Our results suggest new roles for Hh and Wg as instructive polarizing cues that help establish directionality within a cell sheet, and a new polarity-signaling role for the membrane fraction of the oncoprotein Arm.

## Introduction

Cells in multicellular organisms must establish directionality within the plane of a sheet of cells (planar cell polarity or PCP) in order to form complex structures such as organized wing hairs and photoreceptors in *Drosophila* and hair patterns and inner ear epithelia in vertebrates[1]–[4], and yet the signals that initiate PCP remain largely unknown. In vertebrates, Wnt5[5], [6] and Wnt11[7], [8], have been proposed as PCP ligands, but they appear to be permissive rather than instructive since their expression patterns do not coincide with the direction of polarization[4]. In *Caenorhabditis elegans* Wnts have been implicated in cell polarity, both in asymmetric divisions in the early embryo and in the direction of neuronal polarization[9]–[11]. In *Drosophila*, Wnts have largely been excluded from polarity signaling[2], although there is some indication that the canonical Wg and Hh signaling pathways [12], [13] may be involved in determining PCP in the embryonic epidermis[14].

**Figure 1:**
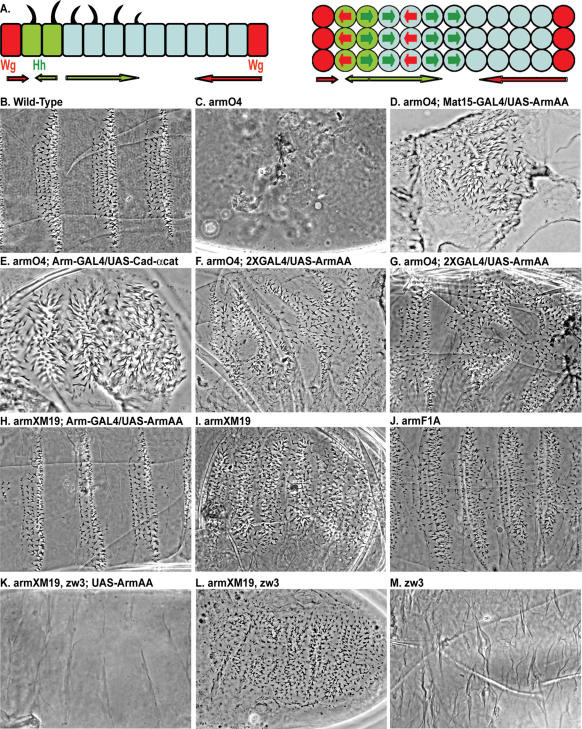
Signaling at the membrane through Arm phosphorylation. A, Schematic of side (left) and top (right) views of one parasegment of the epidermis of a *Drosophila* embryo. Six cells produce denticles; two point anteriorly and four point posteriorly. Hh-producing cells are green, Wg-producing cells are red, and the extent of their signaling domains are shown by the corresponding color arrows. B–M, Phase contrast views of embryos (ventral is up and anterior is left). B, Wild-type denticle pattern. C, *arm^O43A01^* (M/Z) mutant disintegrates due to a lack of epithelial integrity as cellular junctions deteriorate[29], [70]. D, Low levels of expression of Arm^AA^ using the Mat15-GAL4 driver restores some epithelial integrity in an *arm^O43A01^* (M/Z) mutant. E, Strong expression of an α-catenin/E-cadherin fusion protein rescues the *arm^O43A01^* (M/Z) mutant to a similar level (Notice that different drivers were used between D and E to obtain a similar phenotype). F and G, Stronger expression of Arm^AA^ using the Arm-GAL4 and Mat15-GAL4 drivers rescues most epithelial integrity, but leads to disorganization of denticle polarity. Naked cuticle regions (arrows), show activation of Wg signaling, but many denticles point toward or away from the midline, a phenotype not seen in wild-type or other *arm* mutants. H, Expression of Arm^AA^ in *arm^XM19^* (M/Z) mutants leads to an almost wild-type phenotype. I, *arm^XM19^* (M/Z) mutants lose all naked cuticle regions, but retain epidermal integrity and some denticle organization. J, *arm^F1A^* (M/Z) mutants lose naked cuticle, but retain denticle organization. K, Expression of Arm^AA^ in *arm^XM19^*, *zw3* (M/Z) mutants leads to uniform activation of Wg signaling and a completely naked cuticle. L, *arm^XM19^*, *zw3* (M/Z) mutant phenotype is indistinguishable from *arm^XM19^*. M, *zw3* (M/Z) cuticle is naked due to uniform activation of Wg signaling.

Anterior-posterior patterning in the *Drosophila* embryo establishes a segmented body plan[13]. Specification of pattern within segments is controlled by the segment polarity genes, which include the Wnt/Wg and Hh signaling pathways[12]. During late embryogenesis, the epidermis secretes a protective cuticle, which has a repeating pattern of ventral structures known as denticles. Through well-established signal transduction pathways[15]–[17], Wg and Hh instruct cell identity within the embryonic epidermis with Wg directing the naked cell fate and Hh determining the cells that will eventually form denticles. These Actin-rich denticles are structures that display a regular arrangement, with some rows pointing anterior and others pointing posterior (Fig. 1A–B), suggesting a highly organized polarity[12], [14], [18], [19]. The wild-type cuticle pattern consists of 6 rows of cells that secrete Actin protrusions that will become distinct denticles. The shape and size of denticles varies by row[19] and is determined by subsequent EGFR and Notch signaling in later stages[12], [20], [21].

Arm protein is both the major nuclear effector of Wg signaling and a major component of adherens junctions. Adherens junctions provide much of the cell-cell adhesion in epithelia through the transmembrane Cadherin molecules, which establish a physical link between cells. The intracellular domain of Cadherins recruits β-catenin, which in turn recruits α-catenin, thereby linking the transmembrane junctions to the Actin cytoskeleton[22]–[24]. The mechanisms that control the stability of the junction are not well understood. The dissociation of cellular junctions is a process that is directly involved in the transformation of epithelial cells from bound within a cell sheet to migratory or metastatic. Because of this important role in cancer biology, many studies have investigated the link between phosphorylation of β-catenin and the dissociation of adherens junctions especially the roles of the EGF pathway and the oncogene Src (reviewed in [25], [26]). The role of Arm tyrosine phosphorylation, however, remains controversial, since a recent study showed that tyrosine phosphorylation of Arm is dispensable in various developmental processes of *Drosophila* oogenesis[27]. Our findings suggest that there is an *in vivo* requirement for threonine phosphorylation of Arm that regulates Arm at the adherens junction. This threonine phosphorylation is thought to stabilize the interaction between α- and β-catenin, thereby leading to stabilization of cellular junctions[28]. Mutation of these sites increases a cell's migratory potential suggesting that adhesion strength is reduced[28]. CKII is a member of the Wnt signal transduction pathway[1] suggesting that regulation of its activity could regulate threonine phosphorylation of β-catenin allowing for an extracelluloar ligand to modulate adhesion.

The polarized organization of the epidermis of *Drosophila* larvae prompted our investigation of how the Hh and Wg signaling pathways and their components are involved in this polarity. Here we demonstrate that Hh and Wg ligands provide opposing, instructive signals for the orientation of denticles. By genetically manipulating the direction of ligand expression in relatively naïve epithelia, we observe a rotation of polarity consistent with the direction of ligand expression. Further, we find a phosphorylation-dependent role for Arm in the establishment of planar polarity through its function in the polarized subcellular localization of denticle precursors. These results identify new roles for Wg, Hh, and Arm in organizing PCP in embryonic epithelia.

## Results

### Armadillo in junctions and polarity

The classical view of Arm is that it has two non-overlapping functions as a nuclear transcriptional activator of Wg signaling and as a structural component of adherens junctions. These two functions give two different phenotypes in *Drosophila* embryos where loss of junctions leads to cuticle disintegration and loss of nuclear signaling leads to patterning defects (compare Fig. 1 C to I). Many studies, however, have suggested the possibility that Arm's activity at the junction is also actively regulated by signaling, meaning that junctional Arm is not exclusively a static structural component[26]. To test this hypothesis, we mutated two threonines to alanines in Arm (T111A and T121A, hereafter referred to as Arm^AA^) that are required *in vitro* to stabilize its binding to α-catenin[28]. We looked at *Drosophila* cuticles to analyze the effect of Arm^AA^ on both the adherens junctions and on Wg signaling in the nucleus. In strong loss-of-function *arm^O43A01^* maternal and zygotic (M/Z) mutants (hereafter referred to as *arm^O43A01^* (M/Z)) no intact cuticle is made because loss of Arm leads to a drastic loss of cell-cell adhesion in the epidermis. *arm^O43A01^* (M/Z) embryos eventually disintegrate and only small pieces of tissue remain (Fig. 1C). We found that low levels of Arm^AA^ expression restored some cuticular integrity that is completely lost in *arm^O43A01^* (M/Z) embryos, suggesting restoration of some cell-cell adhesion in the epidermis (Fig. 1D)[29].

**Figure 2:**
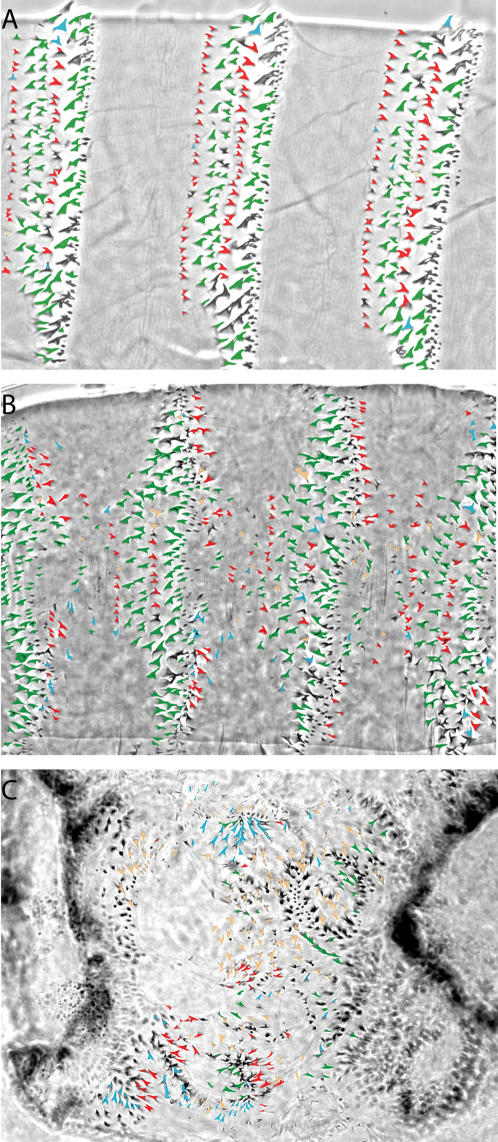
Novel phenotype of Arm^AA^ where the planar organization of denticles is disrupted. A–C, Denticles are colored as follows: those pointing anteriorly are red, posteriorly are green, to the top of the page are blue, and to the bottom of the page are orange. A, Wild-type parasegment showing denticle polarity. Almost no denticles point incorrectly (i.e. those that are colored blue or orange). B, *arm^F1A^* M/Z mutant shows a slight increase in denticles that point incorrectly. C, Approximately half of the denticles in an *arm^O43A01^* (M/Z) mutant expressing Arm^AA^ point incorrectly (i.e. there is an approximately equal number of denticles in each of the four colors).

This phenotype was similar to expression of an α-catenin/E-cadherin fusion protein in *arm^O43A01^* (M/Z) mutants, which restores adherens junctions by bypassing the need for Arm to bridge the α-catenin and E-cadherin interaction. However, expression of this fusion protein does not restore Arm's nuclear signaling function as indicated by a lack of anterior-posterior patterning, or more specifically, a loss of naked cuticle and a lawn of denticles phenotype[30] (Fig. 1D and E). Increasing Arm^AA^ expression levels restored some nuclear Wg signaling activity, as indicated by partial restoration of anterior-posterior patterning, which is shown here by the presence of both naked cuticle and cuticle with denticles (Fig. 1F and 1G). More importantly, increased Arm^AA^ expression revealed a striking new phenotype characterized by the random polarization of denticles (Fig. 2C). Previously observed *arm* loss-of-function phenotypes do not appear to have this level of denticle disorganization (Fig. 1I–J, Fig. 2B, and Table 1). Two pieces of evidence indicated that this denticle disorganization phenotype was due to a lack of functional Arm at the adherens junction, and that Arm^AA^ was competent to transduce the Wg signal in the nucleus. First, in *arm^XM19^* (M/Z) mutants, the truncated Arm^XM19^ protein does not activate the Wg signal in the nucleus, but does retain function in the adherens junction[31]. Expression of Arm^AA^ in *arm^XM19^* (M/Z) mutants lead to an essentially wild-type cuticle in terms of both patterning and denticle organization, (Fig. 1H and I) indicating that Arm^AA^ must fulfill the required role of Arm in transducing the nuclear Wg signal. Second, Arm^AA^ must be competent in transducing the nuclear Wg signal, because a drastic increase in its levels through the removal of the negative Wg pathway regulator *zw3* (in addition to loss of endogenous *arm* function, or an *arm^XM19^, zw3* (M/Z) double mutant) leads to a completely naked cuticle, a hallmark of constitutive Wg signaling activation (Fig. 1K–M)[29]. Therefore, we conclude that phosphorylation of T111 and T121 in Arm is likely to be required for the proper function of Arm at the adherens junction, but not in the nucleus, and perturbation of this phosphorylation leads to disorganized planar polarity of the denticles.

**Table 1:**
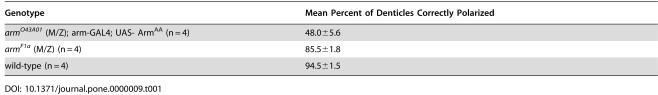
Quantitative Assessment of Denticle Polarity.

Genotype	Mean Percent of Denticles Correctly Polarized
*arm^O43A01^* (M/Z); arm-GAL4; UAS- Arm^AA^ (n = 4)	48.0±5.6
*arm^F1a^* (M/Z) (n = 4)	85.5±1.8
wild-type (n = 4)	94.5±1.5

To further demonstrate that this disorganized denticle phenotype was a defect in planar organization; we compared the orientation of denticles in various genotypes. As described above, wild-type denticles are highly organized and almost always point toward the anterior or posterior of the embryo (Fig. 1B and 2A). Therefore, denticles that point away or toward the midline (the dorso-ventral direction) are mispatterned. We approached this problem both qualitatively by scoring the phenotype (Fig. 2 shows denticles colored according to their orientation with blue and orange representing D/V oriented denticles; Table 2: 1–12 offers a summary of various genotypes scored qualitatively) and quantitatively by counting denticles that are mispolarized (Table 1). These experiments showed that in wild-type and weak *arm* loss-of-function mutants, most denticles point correctly (94.5±1.5% and 85.5±1.8% respectively). In contrast, in *arm^O43A01^* (M/Z) embryos expressing Arm^AA^, only about half of the denticles point correctly (48.0±5.6).

**Table 2:**
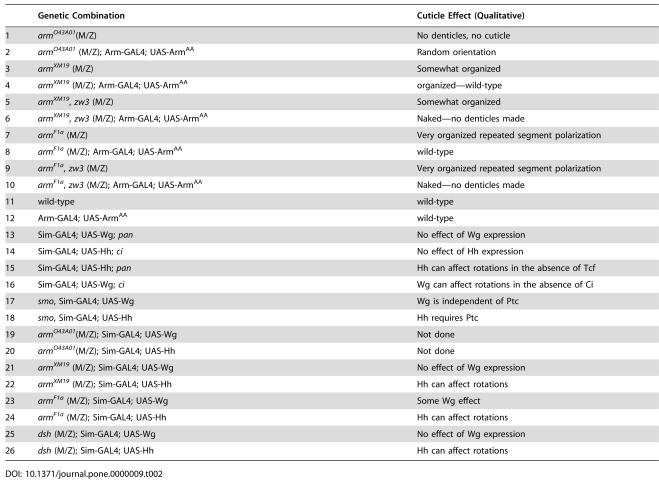
Qualitative assessment of denticle polarity phenotypes.

	Genetic Combination	Cuticle Effect (Qualitative)
1	*arm^O43A01^*(M/Z)	No denticles, no cuticle
2	*arm^O43A01^* (M/Z); Arm-GAL4; UAS-Arm^AA^	Random orientation
3	*arm^XM19^* (M/Z)	Somewhat organized
4	*arm^XM19^* (M/Z); Arm-GAL4; UAS-Arm^AA^	organized—wild-type
5	*arm^XM19^*, *zw3* (M/Z)	Somewhat organized
6	*arm^XM19^*, *zw3* (M/Z); Arm-GAL4; UAS-Arm^AA^	Naked—no denticles made
7	*arm^F1a^* (M/Z)	Very organized repeated segment polarization
8	*arm^F1a^* (M/Z); Arm-GAL4; UAS-Arm^AA^	wild-type
9	*arm^F1a^*, *zw3* (M/Z)	Very organized repeated segment polarization
10	*arm^F1a^*, *zw3* (M/Z); Arm-GAL4; UAS-Arm^AA^	Naked—no denticles made
11	wild-type	wild-type
12	Arm-GAL4; UAS-Arm^AA^	wild-type
13	Sim-GAL4; UAS-Wg; *pan*	No effect of Wg expression
14	Sim-GAL4; UAS-Hh; *ci*	No effect of Hh expression
15	Sim-GAL4; UAS-Hh; *pan*	Hh can affect rotations in the absence of Tcf
16	Sim-GAL4; UAS-Wg; *ci*	Wg can affect rotations in the absence of Ci
17	*smo*, Sim-GAL4; UAS-Wg	Wg is independent of Ptc
18	*smo*, Sim-GAL4; UAS-Hh	Hh requires Ptc
19	*arm^O43A01^*(M/Z); Sim-GAL4; UAS-Wg	Not done
20	*arm^O43A01^*(M/Z); Sim-GAL4; UAS-Hh	Not done
21	*arm^XM19^* (M/Z); Sim-GAL4; UAS-Wg	No effect of Wg expression
22	*arm^XM19^* (M/Z); Sim-GAL4; UAS-Hh	Hh can affect rotations
23	*arm^F1a^* (M/Z); Sim-GAL4; UAS-Wg	Some Wg effect
24	*arm^F1a^* (M/Z); Sim-GAL4; UAS-Hh	Hh can affect rotations
25	*dsh* (M/Z); Sim-GAL4; UAS-Wg	No effect of Wg expression
26	*dsh* (M/Z); Sim-GAL4; UAS-Hh	Hh can affect rotations

**Figure 3:**
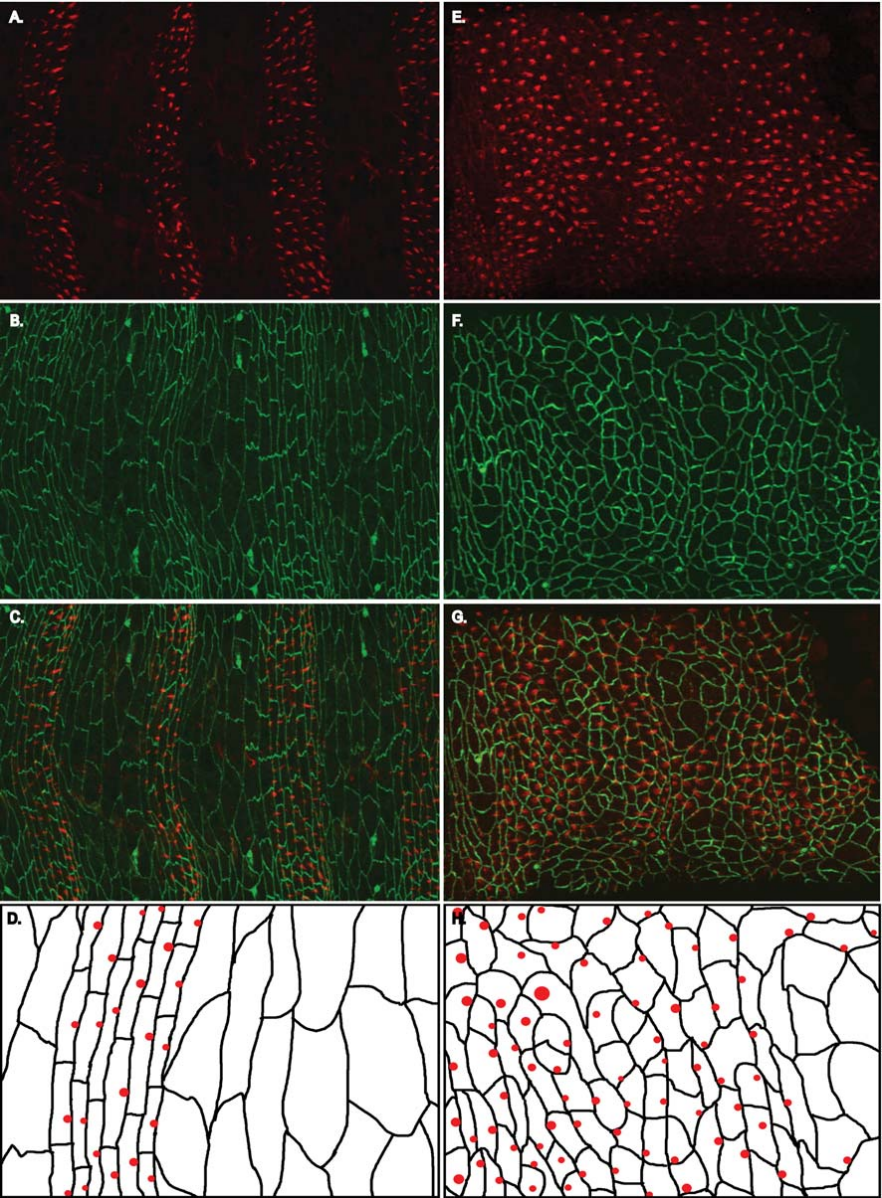
The precursors of denticles, the Actin foci, are mislocalized in embryos expressing Arm^AA^, and the asymmetric distribution of Arm is disrupted. A–D, wild-type. E–H, *arm^O43A01^* (M/Z) mutant expressing Arm^AA^. A, Actin staining shows the Actin accumulation that is the precursor to denticle formation. B, In wild-type embryos, Arm localizes to the D/V boundaries of the smaller, rectangular-shaped cells that will produce denticles. C, Overlay of Arm and Actin staining shows that the denticle-producing cells are smaller and rectangular. D, Schematic of a parasegment showing that the cells that produce denticles are smaller and rectangular than the cells that do not produce denticles and that the Actin foci that will become denticles (red dots) are located on the posterior margin of the cells. Some cells produce more than one Actin focus, but only one shown per cell for simplicity in the schematic. E, Actin staining shows that most cells produce denticle precursors, though some cells do not. F, Arm staining shows that the stereotypical cell-shape changes are impaired, and the asymmetric distribution of cell membrane Arm is disrupted. G, Overlay of Arm and Actin staining shows that the denticle precursors are mislocalized. H, Schematic showing the apparent random localization of denticle precursors (red dots).

To determine the cell biological basis of this defect, we investigated the epithelia that will produce denticles. Immunofluorescence studies during late embryogenesis revealed that in wild-type embryos, ventral epidermal cells that will produce denticles adopt different cell shapes than those that will produce naked cuticle. Denticle-producing cells are small and rectangular, whereas naked cuticle cells are large and amorphous. Further, the Actin foci that will eventually become denticles are almost always localized to the posterior margin of the cell (Fig. 3A–D, and [14]). Interestingly, we found that Arm is asymmetrically localized predominantly to the dorsal or ventral (D/V) sides of the denticle-producing cells (Fig. 3B and [14]). In Arm^AA^ embryos, the Actin foci are localized randomly (Fig. 3E) and the D/V polarization of Arm is disrupted (Fig. 3F). Taken together, the denticle disorganization, the failure of epidermal cells to take on the correct shapes, the improper asymmetric membrane polarization of Arm, and the failure of Actin foci to be asymmetrically localized to the posterior margin of the epidermal cells in Arm^AA^ expressing embryos indicates that threonine phosphoryration of Arm is likely to be required for proper planar polarization in the epidermis.

### Wg and Hh signaling in polarity

**Figure 4:**
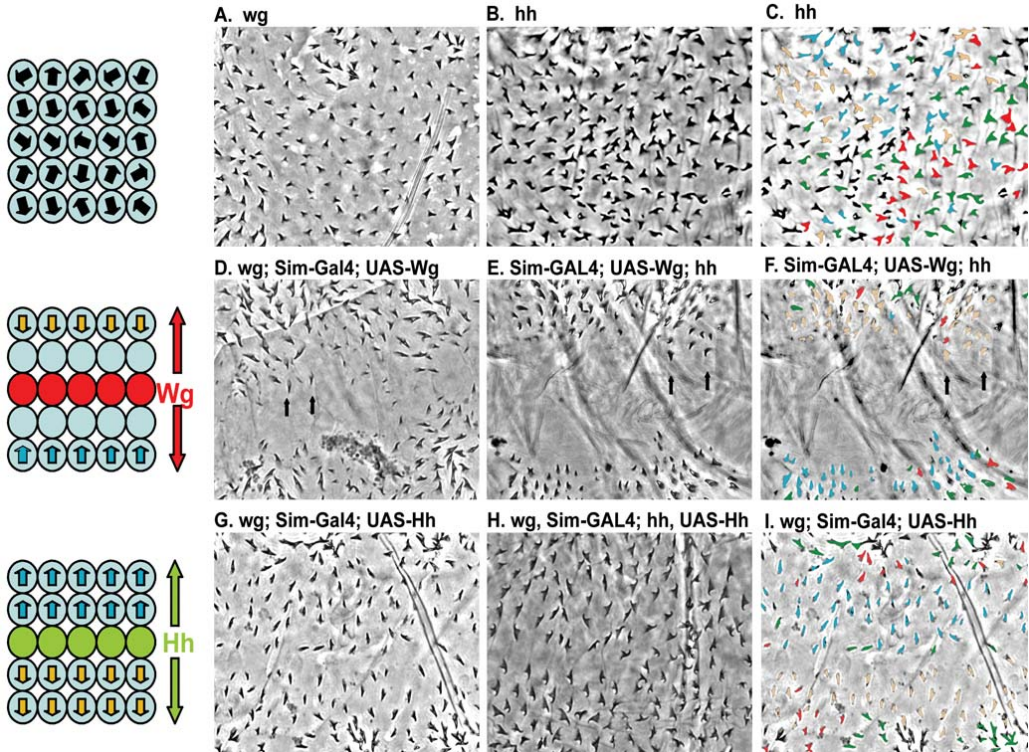
Wg and Hh act as instructive signals for the orientation of denticles in the *Drosophila* embryonic epidermis. (Left top) Schema depicting wild-type epithelia with denticle polarity direction depicted by black arrows. (Left middle) Schema showing that Wg (red cells) expression along the ventral midline transforms cells to naked cell-fate, and leads bordering cells to rotate their denticles toward the source of Wg. (Left bottom) Hh (green cells) expression does not transform the cell-fate, but leads to denticles orienting away from its source. A–I, Phase contrast views of embryos (ventral is up and anterior is left). A, *wg* and B, *hh* mutants display relatively random planar polarity and the ventral lawn of denticles phenotype (see[19] for a detailed explanation of denticle cell fates). C, Same image as in B, but with denticles colored according to orientation, as in Fig. 2. Note the relatively random distribution of colors. D and E, Ectopic expression of Wg in a stripe perpendicular to its normal pattern leads to a reorientation of denticles toward the source of Wg in both *wg* and *hh* mutants (arrows). F, Same image as in E, but with the denticles colored according to orientation. Note that the blue and orange denticles line up and point toward the ventral midline, or toward the source of Wg. G and H, Expression of Hh in a stripe perpendicular to its normal pattern leads to a reorientation away from the source of Hh in both *wg* and *wg; hh* double mutants. I, Same image as in G, but with the denticles colored according to their orientation. Note that the blue and orange denticles line up in a pattern directly opposite to that observed in F showing that the denticles near the midline orient away from the source of Hh ligand.

**Figure 5:**
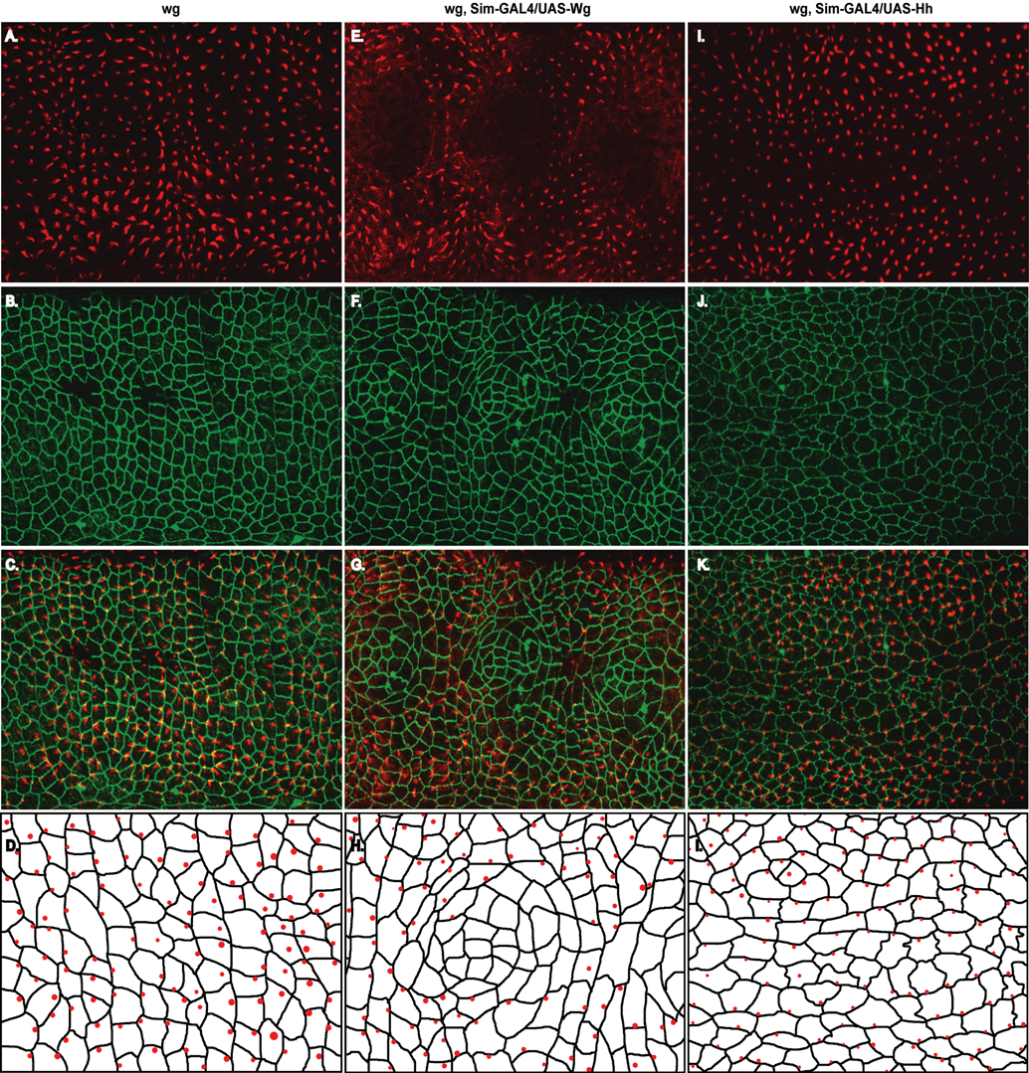
Wg and Hh can relocalize the Actin foci that are denticle precursors. A–D, *wg*. E–H, *wg*, Sim-GAL4/UAS-Wg. I–L, *wg*, Sim-GAL4/UAS-Hh. C, Overlay of A, Actin and B, Arm staining shows that the stereotypical cell-shape changes are impaired and that all cells produce denticles. D, Schematic showing the apparent random localization of denticle precursors (red dots). E, Actin staining showing that expression of Wg along the ventral midline causes naked cuticle to form in some cells along the midline. G, Overlay of E, Actin and F, Arm staining shows apparent cell-shape reorganization in cells that are transformed to the naked cell fate along the midline. In most cells that border the cells that lack denticle precursors, the Actin foci are relocalized to the D/V margin of the cell. H, Schematic showing the D/V localization of denticle precursors in cells that border the naked cells along the midline. K, Overlay of I, Actin and J, Arm staining shows that in at least 8–9 rows of cells surrounding the ventral midline, the denticle precursors are consistently relocalized to the D/V margins of cells. L, Schematic showing that in cells receiving the Hh signal (i.e. those surrounding the midline), cell shapes are different than in the *wg* mutant alone and that the denticle precursors (red dots) are relocalized to the D/V margins of cells.

In order to test whether the denticle polarity phenotypes in Arm^AA^ expressing embryos were the result of intercellular signaling, we turned to the signaling molecules Wg and Hh for several reasons. First, in both *wg* and *hh* mutants, all ventral cells make denticles, but their planar polarity is clearly disrupted (Fig. 4A–C). Instead of pointing anterior or posterior, many of the denticles point toward or away from the midline. Second, the cell shape changes that distinguish denticle producing cells from naked cuticle cells do not occur in *wg* and *hh* mutants, as all cells appear roughly square (Fig. 5B). Lastly, Wg and Hh are normally expressed in two anterior/posterior (A/P) cell stripes, which coincide with the direction of denticle polarity suggesting the possibility that they act as directional cues.

Since Wg and Hh are required for proper cuticle organization[14], we tested whether Wg and Hh are sufficient instructive cues for planar polarity of denticles. To accomplish this, we eliminated endogenous *wg* and expressed Wg in a stripe along the ventral midline, which is perpendicular to its normal expression pattern. Since *wg* and *hh* function in an autoregulatory loop in the embryonic epidermis, removal of one leads to the absence of both in late stages[12]. Strikingly, we observed a rotation of denticles toward the midline, or toward the cells that are ectopically expressing Wg (Compare Fig. 4D to 4A). The rotation of the denticles toward Wg was only partial in the *wg* mutants. We postulated that this may be due to the effect of Wg signaling on Hh expression[12]. Normally Wg is required for Hh expression, so expression of Wg along the ventral midline likely leads to inappropriate activation of Hh expression. To address this, we expressed Wg along the ventral midline in *hh* mutant embryos. In this situation, the denticles rotate toward the ventral midline en masse (Fig. 4E and F), suggesting that Wg and Hh may compete in instructing denticle polarization. Since Wnt molecules have previously been implicated in planar cell polarity (PCP) signaling[4], the effect of Wg on denticle polarity was not entirely unexpected, however Hh's effect on polarity was surprising since the Hh signaling pathway has not been implicated in PCP signaling[32]. Since Hh appeared to be modifying the effect of Wg on denticle orientation, we asked whether expression of Hh along the ventral midline in a *wg* mutant embryo would have an effect on polarity. Surprisingly, the denticles rotated away from the midline, or away from the cells that were ectopically expressing Hh (Fig. 4G and I). We also expressed Hh at the ventral midline in a *wg* and *hh* double mutant, and found that the denticles still rotate away from the midline, or away from the cells that are ectopically expressing Hh (Fig. 4H). In these experiments, the expression of Wg and Hh was rotated 90° from their normal expression patterns. Our finding that the denticles rotate by a similar degree indicates that Wg and Hh are sufficient to instruct denticle rotation and therefore suggests that they act as directional cues for the planar cell polarity of epithelial cell sheets on a gross scale.

We next investigated the effects of Wg and Hh on the sub-cellular localization of the Actin foci that eventually become denticles. In *wg* and *hh* mutants, these Actin foci are no longer restricted to the posterior margin of epidermal cells (Fig. 5 A–C and [14]). Although sometimes they are correctly localized to the posterior margin, these Actin foci are also found at the anterior margin as well as on the dorsal and ventral margins of cells in *wg* mutants.

When we expressed Wg along the ventral midline in wg mutants, we saw that the Actin foci were no longer found in many cells surrounding the midline, consistent with our cuticle analysis results. We presumed that the cells lacking the Actin foci must be changing fate in response to the Wg signal, and secreting naked cuticle. The cells of the next row, or those bordering the cells lacking the Actin foci, were most likely receiving the Wg signal too as they were in a similar position to cells in denticle row 1 (Fig. 1A, first green cell). In the wild-type epidermis, only the first cell in the denticle-forming stripe of cells receives the Wg ligand due to a segment boundary that is established between the first and second (most anterior or two green cells in Fig. 1A) denticle-producing cells[33]. Importantly, this corresponds to the orientation of the most anterior denticle, which points toward the source of Wg (see Fig. 1A). In embryos that express Wg along the ventral midline, many of the cells that border the cells lacking the Actin foci contain Actin foci that are no longer localized to the posterior margin. In fact, many of them appeared to be localized to the dorsal or ventral margin of the cell, suggesting that the foci had rotated 90° in response to the Wg signal (Fig. 5D–F). We next investigated the effect of Hh on the subcellular localization of the Actin foci. We found that when we expressed Hh in a stripe along the ventral midline, we saw a drastic rotation of the Actin foci localization. We found that several rows of cells at the midline consistently had the Actin foci localized to either the dorsal or ventral margin (Fig. 4D). These results taken together suggest a molecular mechanism whereby Wg and Hh can direct the localization of Actin foci that precede denticle formation and thus direct the polarity of denticles.

### Wg and Hh pathway components in denticle polarity signaling

**Figure 6:**
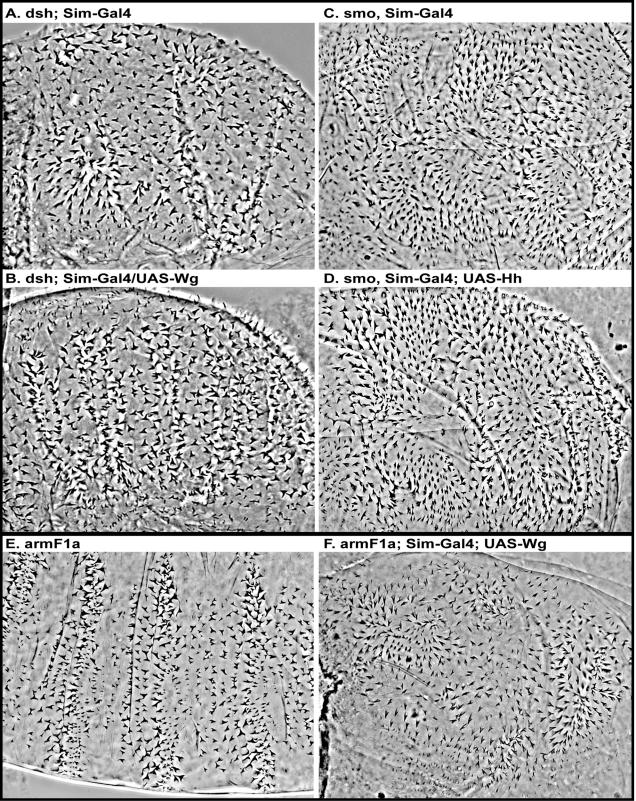
Smo, Dsh and Arm are required for polarity signal transmission. A, A *dsh* ((M/Z)) mutant embryo showing a lawn of randomly oriented denticles. B, Expression of Wg along the ventral midline does not affect denticle orientation in embryos that are mutant for *dsh*. C, A *smo* (M/Z) mutant embryo also shows a lawn of denticles phenotype, and a loss of planar polarization. D, Expression of Hh along the ventral midline in *smo* mutant embryos has no observable effect on the planar polarization of denticles, or on the lawn of denticles phenotype. E, *arm^F1A^* (M/Z) mutant embryos show a denticle-covered cuticle phenotype, but some patterning is preserved due to a low level of Wg nuclear signaling activity retained by this allele[29]. F, Expression of Wg along the ventral midline in some cases leads to a disruption of the patterning observed in *arm^F1A^* M/Z mutants alone, and causes mispolarization of denticles.

When Hh binds to its receptor, Patched (Ptc), repression of the Hh pathway activator Smoothened (Smo) is relieved, thereby activating the Hh pathway[34], [35]. To test the involvement of other Hh pathway components in denticle polarization, we generated *smo* (M/Z) mutant embryos that expressed Hh along the ventral midline. As expected for standard Hh signal transduction, in the absence Smo, Hh cannot affect the rotation of denticles because the Smo/Ptc receptor complex is compromised (Fig. 6D)[13].

To assess the involvement of other Wg pathway components in denticle polarization, we made *disheveled (dsh* M/Z*)* null mutant embryos and expressed Wg along the ventral midline. In Wg signaling, the Dsh protein is required for the transmission of Wg signal within cells, and acts in both the PCP and canonical Wnt pathways. Embryos lacking Dsh resemble *wg* mutants in both the lack of naked cuticle and the lack of denticle organization (Fig. 6A). Expression of Wg in *dsh* mutants did not lead to any observable changes in denticle orientation or cell-fate transformation (Fig. 6B) showing that Dsh is required for Wg-dependent denticle organization.

Next, we examined the involvement of downstream transcription factors in denticle polarity. The transcription factor TCF (*pangolin* or *pan*) is required for Wg signaling in the nucleus. In the absence of TCF (*pan* mutants), expression of Wg along the ventral midline had no visible effect on the polarity of the denticles (results summarized in Table 2). We also tested the role of the Hh transcription factor *cubitus interruptus* (*ci* or Gli). When we expressed Hh along the ventral midline in *ci* mutants, we observed that Hh caused no visible effects. These results taken together suggest that both the Hh and Wg ligands require a transcriptional response in order to affect denticle polarities.

Finally, we investigated whether Arm is required for the transmission of the Wg polarity signal. We approached this by using an allelic series of *arm* mutants expressing Wg along the ventral midline. The strongest allele, *arm^O43A01^*, does not make cuticle, so the experiment wasn't attempted. The next allele, *arm^XM19^*, is unable to transmit the Wg signal due to its low levels, but it retains junction function since the cuticle remains intact[29], [31]. However, expression of Wg along the ventral midline in this mutant had no observable effect on denticle orientation (data not shown) likely due to overall low protein levels [31]. The weakest allele, *arm^F1a^*, is the most useful, because it loses most of its ability to function as a transcriptional activator (shown here by loss of naked cuticle), though mutant protein levels are high and junctions appear normal[36]. In *arm^F1a^* (M/Z) embryos expressing Wg along the ventral midline, we observed some reorientation of denticles toward the midline (compare Fig. 6E to F). This allelic combination allows the observation of Wg effects on Arm protein at the junction, because Arm protein is present in adequate levels, but all cells make denticles due to this point mutant's inability to activate Wg transcriptional targets. This phenotype showed a weak penetrance (∼10% of embryos when ∼50% was expected, n>500) likely due to the competition between normally expressed Wg which is maintained in the *arm^F1a^* mutant[36] and the ectopic form which was not strongly expressed under our experimental conditions. These results suggest that the ligand Wg can also affect the orientation of denticles without acting through the nuclear Wg signaling pathway and affecting the cell fate decision, since no new patches of naked cuticle are observed at the midline. Interestingly, these results suggest that the effect on denticle polarity of Wg in the *arm^F1a^* (M/Z) mutants may be through the membrane pool of Arm at the adherens junctions. Taken together, our results suggest that proper PCP in the epidermis requires both a transcriptional response of Wg and Hh and thereby a functional Arm in the nucleus, as well as a functional Arm at adherens junctions.

## Discussion

Our results indicate that Wg and Hh act as instructive cues in the *Drosophila* embryonic epidermis to establish planar cell polarity. Though the complete molecular mechanisms that control the complex system of PCP in the ventral epidermis remain to be determined, this process appears to occur in part through the asymmetric localization of Arm at the membrane. Further, proper polarity signaling is abolished if specific phosphorylation sites within the α-catenin binding domain of Arm are mutated. These sites were originally found to increase the affinity of β-catenin for α-catenin when phosphorylated by Casein Kinase II *in vitro*, suggesting one mechanism for stabilizing junctions[28]. Our findings provide *in vivo* support for this hypothesis, as low levels of Arm^AA^ rescued cellular junction defects to a similar extent as expression of an α-catenin/E-cadherin fusion protein, a protein that makes overly stable junctions [30]. Higher levels of Arm^AA^ expression lead to apparent polarity defects. As Arm^AA^ does not localize asymmetrically the way that wild-type Arm does, we inferred that CKII phosphorylation may be required for the accumulation of junctions in specific regions of cells implying that stable junctions at specific sites in a cell are required for proper planar cell polarity. Further, our findings revealed that when all signaling activity is abolished through null mutations in the Wg or Hh signaling pathways, both cell identity and polarity determination was disrupted. It remains to be determined how Wg and Arm proteins function in polarity signaling, specifically whether they work through known PCP components, function similarly to their role in dorsal closure, or perhaps through novel signaling mechanisms like the interaction with Notch or Axin[36]–[39].

The *wg* and *hh* genes are required for the proper establishment of cell identities within segments[12]. Several studies have suggested that there are multiple roles for Wg and Hh during embryogenesis[40]–[44]. Uniform expression of Wg in the embryo leads to a completely naked cuticle[45], but short early bursts of expression establish what appears to be relatively normal patterning[44]. Upon closer inspection, however, the denticle orientations of these early expression rescue experiments do not entirely resemble the wild-type patterning[44], [46]. This suggests that early expression of Wg can rescue several aspects of cell identity, including development of naked cuticle, but Wg is also required in the later stages when denticles form to specify proper orientations. Expression of ectopic Wg has been observed to correlate with denticles pointing toward the source of Wg[47], and expression of ectopic Hh also leads to denticles pointing away from its source[48]. These studies, however could not distinguish between cell fate transformation and changes in cell polarity since the sources of both ligands were in the normal orientation. Our observations argue that Hh and Wg can have direct effects on cell polarity since denticles and their precursors (the Actin foci) are rotated 90° away from the anterior-posterior axis corresponding to the direction of ligand expression.

In the early embryo, expression of Wg and Hh is determined by pair-rule genes, but this effect is transient and requires mutually reinforcing positive activation loops to form between cells expressing Wg and En/Hh[12]. This is the early signaling event that establishes an organizer region in each parasegment[48]. Therefore, if either Hh or Wg is missing, expression of both is lost. The early effects of Hh and Wg expression are important for the establishment of segment boundaries[47], [49], and these boundaries function in limiting Wg function, giving this morphogen an asymmetric range[33]. Our findings agree with these observations, because we observe that the Wg effect is best observed when *hh* is absent, suggesting that when the *hh* gene is present a boundary may be formed, thus preventing Wg from orienting the denticles to the same extent. It also appears that the distance over which Wg can act is longer in the absence of *hh* as expected from previous observations[33]. According to the proposed boundary model, the extent of Wg influence is to the first denticle-secreting cell, but not beyond[33]. This finding, along with our discovery that denticles orient toward the source of Wg, may explain why the first row of denticles in wild-type larvae points toward the anterior of the embryo. Only this row of cells receives Wg signal as the segment boundary blocks further action by Wg to the next row of cells[33]. On the other hand, Hh can and does affect the next two rows of cells. We found that expression of Hh causes a rotation away from the source, and could explain why the next two rows of denticles point toward the posterior of the embryo. Our results do not explain the final orientation of all rows of denticles, and one likely complication is that in late embryonic stages the Notch and EGFR signaling pathways affect the identities of cells within the denticle belt[12], [20], [21]. It will be interesting to test what effects these signals have on the final orientation of the orientation of denticles, and whether the Notch pathway functions in polarity as well.

The PCP signaling pathway determines planar polarity in a variety of tissues[4]. In vertebrate and *C. elegans* studies, Wnts have been implicated in the establishment of polarity, but only one study in *Drosophila* suggested a role for Wg in PCP[14]. In fact, the present model excludes the known morphogens, and suggests that PCP is established through cell-cell interactions involving atypical cadherins like Flamingo or through an as yet unidentified factor X[50]–[57]. Though our study does not address the function of the known components of PCP signaling in the embryo, it is interesting that mutants in PCP signaling pathway components affect the polarity of the first two rows of denticles[14], [58]. Our findings support the possibility that Wg and Hh lead to the expression of an unknown factor affecting the polarization of denticles, because blocking the transcriptional readout of either Wg or Hh with *tcf* or *ci* mutations respectively prevents the polarizing activity of both pathways. This is similar to the PCP disruptions found in the *Drosophila* eye model for Wg signaling components[50]. Our observations do, however, offer a further possibility, namely that by blocking all Wg signaling with null mutations the underlying polarity organizing function of Wg may be obscured. We find that in the weak *arm^F1A^* mutant the orientation of denticles can be affected by the expression of Wg without affecting the cell-fates, suggesting that perhaps Wg can affect polarity directly. This effect of Wg was not observed in stronger *arm* mutant embryos suggesting that Arm protein is required for the Wg effect on denticle orientation. Interestingly, cell culture work has recently implicated Wg in controlling adherens junction strength[59].

The use of the embryonic epidermis allowed us to observe the interesting possibility that Arm functions in cell polarity. Since some of the molecules involved in the PCP signaling pathway are similar to Cadherins[57], it seems logical that adhesion is involved in the establishment of polarity. However, adherens junctions have not been implicated so far. This is likely due to the difficulty of working with adherens junction component mutations that are often cell-lethal in the systems that have been used to study PCP. Here we have used the embryo, a system that allows relatively simple perturbation of *arm* function, and efficient ubiquitous or directional ectopic expression. Unfortunately, the major limitation of the ventral midline expression assay is that it only works for secreted, diffusible ligands. Thus, cell-autonomous activation of Hh or Wg pathway components (such as with activated Arm or Smo) along the ventral midline cannot be observed, since these cells invaginate and do not become a part of the external epidermis. We are currently working on ways to overcome this technical limitation.

The fact that β-catenin is both an oncogene and a component of adherens junctions has led to many studies attempting to link the phosphorylation state of β-catenin in adherens junctions to the epithelial to mesenchymal transition (EMT) in cancer cells and during development. Phosphorylation of tyrosine residues in β-catenin is thought to lead to disassembly of adherens junctions[25], [26], [60], but recent studies both *in vivo* and *in vitro* have challenged this[27], [61]. Certainly these discrepancies will have to be resolved, but here we provide evidence for a different mechanism for regulating junctions, and perhaps EMT, through threonine phosphorylation-based stabilization or dephosphorylation-based destabilization of junctions. It will be crucial to establish which is the regulated step, and whether there are any phosphatases involved in this process in addition to the known kinase CKII[28].

Interestingly, the recent findings that α-catenin and β-catenin do not form a stable complex in junctions [23], [24], suggests a possible explanation for our findings. We speculate that expression of Arm^AA^ can rescue the basic activity of junctions lost in strong *arm* mutant embryos, which is to hold a tissue together. However, its reduced affinity for α-catenin does not cause a local increase in α-catenin levels and therefore Actin levels do not become asymmetric. This leads to a skewing of the normal polarization of the Actin cytoskeleton. It will be crucial to determine how junctions are localized asymmetrically in the first place, and whether this is dependent on extracellular signaling. These findings, and the effects of α-catenin mutations on inflammation and tumor progression in the mouse epidermis[62] make analysis of the interaction between α- and β-catenin particularly important.

These experiments provide some of the first evidence that the Hh signaling pathway is involved in polarity. It is particularly interesting that Hh expression leads to the reorganization of Actin structures within epithelial cells, since this suggests that Hh can affect the polarity of the Actin cytoskeleton. This finding is also relevant to cancer biology, because during metastasis, cancer cells lose polarity and essentially ignore their environment. Our results show that Wnts and Hh can affect cell polarity, in addition to their well-known effects on cell proliferation[16], [63], [64]. Along with the recent report that TGFβ signaling affects polarity and EMT[65], our findings imply that this dual role may be a general feature of oncogenic signaling pathways.

## Methods

### 

#### Constructs and Flies

Generation of germline clone embryos from flies with *arm* mutations *arm^O43A01^*, *arm^F1A^*, *arm^XM19^*, and *arm^XM19^*, *zw3^M11-1^* double mutant are described in detail[29], [66]. For a description of *wg^CX4^, pan^2^, ci^Ce2^, hh^3^, smo^2^, ptc^IN108^* and *dsh^V26^* see Flybase (http://flybase.bio.indiana.edu/).

Maternal and zygotic mutant eggs were generated by the dominant female sterile technique [67]. For all expression experiments, the Arm-GAL4 embryonic ubiquitous driver, the mat15-Tub-Gal4[68] maternally contributed driver, and Sim-GAL4 for specific ventral midline expression were used. None of these drivers cause phenotypes on their own, and in all genetic experiments non-expressing siblings were used as the wild-type controls. The Sim-GAL4 driver proved to be rather weak, because expression of UAS-Wg and UAS-Hh in a wild-type background showed only very mild effects (not shown).

The Arm^AA^ construct was generated with T111A and T121A mutations through site-directed mutagenesis with primers 5′-CCGGAAGCCCTGGAGGAGGGCATTGAGA TTCCCTCCGCCCAGTTTGAT and its complement (Quickchange from Stratagene), and fused to an N-terminal double HA tag in pUASt[69]. These sites correspond to T102 and T112 in human β-catenin. We tested four independent transgenic lines that behaved indistinguishably, and used two separate insertions on two different chromosomes to complete these experiments. In our hands, expression of this Arm transgene in embryos had no visible effect when endogenous *arm* allele was wild-type. This results from the fact that only stabilized forms of Arm can overcome the degradation machinery and give phenotypes in the embryo[29].

#### Antibodies and Embryo Fixations

The HA 3F10 rat antibody was from Roche. Anti Armadillo, Wingless, Patched, Sexlethal, Actin, and E-Cadherin antibodies were obtained from the Developmental Studies Hybridoma Bank developed under the auspices of the NICHD and maintained by The University of Iowa, Department of Biological Sciences, Iowa City, IA 52242. Fixations, stainings, and cuticle preps as described previously[29]. Actin, HA, Sxl and Arm stainings were performed on heat-fixed embryos. Though not shown, the Sexlethal antibody was used to sex embryos. This allows for the identification of male embryos laid by germline clone mothers, which are hemizygous and therefore maternally and zygotically mutant for X-chromosome genes. To detect the expression domains of Hh, we used the Ptc antibody and the Wg antibody to detect Wg expression domains (Not shown in figures). Images were acquired on a Zeiss Axioimager with Apotome. Image processing was done with Volocity (Improvision), Photoshop and Illustrator (Adobe).

#### Denticle Rotation Analysis

40× phase contrast images of cuticles were analyzed for denticle polarity by eye in Adobe Photoshop. Cuticles were oriented such that anterior was to the left and posterior was to the right. Denticles that appeared to within 45 degrees to the right or to the left of exactly anteriorly, posteriorly, up, and down were pseudo-colored red, green, blue, and orange respectively. The very tip of the denticle showing the characteristic hook of each denticle was a useful marker in deciding in which direction the denticle was pointing. For quantitation of wild-type, *arm^F1A^* (M/Z), and *arm^O43A01^* (M/Z) mutants expressing Arm^AA^, 300–600 denticles were counted in each embryo (n = 4/genotype). Denticles that were pointing anteriorly or posteriorly were classified as denticles that were pointing correctly, while denticles that were pointing up or down were classified as denticles that were pointing incorrectly. We counted any denticle that was able to be scored within the field of view. Any denticle whose polarity was obscured or not in focus was left unscored in all images.
